# Combination of Irreversible Electroporation and STING Agonist for Effective Cancer Immunotherapy

**DOI:** 10.3390/cancers12113123

**Published:** 2020-10-26

**Authors:** Eun-Jin Go, Hannah Yang, Hong Jae Chon, DaSom Yang, WonHyoung Ryu, Dong-Hyun Kim, Dong Keun Han, Chan Kim, Wooram Park

**Affiliations:** 1 Department of Biomedical-Chemical Engineering, The Catholic University of Korea, 43 Jibong-ro, Wonmi-gu, Bucheon-Si, Gyeonggi-do 14662, Korea; gej8728@chauniv.ac.kr; 2 Department of Biomedical Science, CHA University, 335 Pangyo-ro, Bundang-gu, Seongnam-si, Gyeonggi-do 13488, Korea; 3 Medical Oncology, CHA Bundang Medical Center, CHA University School of Medicine, 59 Yatap-ro, Bundang-gu, Seongnam-si, Gyeonggi-do 13496, Korea; hnyang23@gmail.com (H.Y.); minidoctor@cha.ac.kr (H.J.C.); 4 Department of Mechanical Engineering, Yonsei University, Seoul 03722, Korea; ds.yang@northwestern.edu (D.Y.); whryu@yonsei.ac.kr (W.R.); 5 Department of Radiology, Feinberg School of Medicine, Northwestern University, Chicago, IL 60611, USA; dhkim@northwestern.edu; 6 Robert H. Lurie Comprehensive Cancer Center, Chicago, IL 60611, USA; 7 Department of Biomedical Engineering, McCormick School of Engineering, Evanston, IL 60208, USA; 8 Department of Bioengineering, The University of Illinois at Chicago, Chicago, IL 60607, USA

**Keywords:** cancer immunotherapy, irreversible electroporation (IRE), stimulator of IFN genes (STING) agonists, immunogenic cell death (ICD), combinational treatment

## Abstract

**Simple Summary:**

This study deals with a new strategy for effective cancer immunotherapy using a combination of electrical ablation and immune adjuvant, a stimulator of interferon genes (STING) agonist. The combination treatment significantly improved the cancer treatment effect by converting the immunosuppressive tumor microenvironment (TME) to an immunogenic TME. The combination of interventional oncology and immuno-oncology is expected to contribute to the treatment of various difficult-to-treat tumors.

**Abstract:**

Recently, cancer immunotherapy has received attention as a viable solution for the treatment of refractory tumors. However, it still has clinical limitations in its treatment efficacy due to inter-patient tumor heterogeneity and immunosuppressive tumor microenvironment (TME). In this study, we demonstrated the triggering of anti-cancer immune responses by a combination of irreversible electroporation (IRE) and a stimulator of interferon genes (STING) agonist. Optimal electrical conditions inducing damage-associated molecular patterns (DAMPs) by immunogenic cell death (ICD) were determined through in vitro 2D and 3D cell experiments. In the in vivo syngeneic lung cancer model, the combination of IRE and STING agonists demonstrated significant tumor growth inhibition. We believe that the combination strategy of IRE and STING agonists has potential for effective cancer immunotherapy.

## 1. Introduction

Cancer immunotherapy using immune checkpoint inhibitors harnessed the host immune system to elicit potent and durable anti-cancer immune responses and revolutionized the treatment landscape of advanced cancers [1]. The efficacy of cancer immunotherapy is dependent on the optimal activation of CD8^+^ T cells during the cancer-immunity cycle [2]. In the initial stages of this cycle, tumor antigens should be released from tumor cells and cross-presented to CD8^+^ T cells by dendritic cells in order for tumors to be appropriately recognized by the host immune system [3]. However, most tumors lack adequate tumor antigen presentation, evade host immune recognition, and therefore generate a poorly immunogenic tumor microenvironment that harbors intrinsic resistance to cancer immunotherapy [4,5]. To overcome these limitations, multidisciplinary researchers are actively investigating effective approaches to reinforce the innate immune sensing of tumor cells and trigger a robust adaptive immunity against cancer [6,7,8,9,10,11].

Irreversible electroporation (IRE) is a minimally invasive treatment modality for localized tumors [12,13,14]. Electrical stimuli cause apoptosis of cancer cells by inserting an electrode needle directly into the tumor mass and repeatedly applying short electrical pulses at high voltage to induce permanent damage to the cellular membrane [15,16,17]. It was recently reported that IRE could induce immunogenic cell death (ICD), showing its potential for use in cancer immunotherapy [18,19,20]. Tumor antigens are also released from dying tumor cells after electrical stimulation. As a result, antigen-presenting cells (APCs) such as dendritic cells (DCs) can be activated, producing prime CD8^+^ cytotoxic T cell responses against cancer cells. Additionally, since IRE is non-thermal ablation, thermal damage can be minimized compared to conventional thermal ablative technologies such as radiofrequency ablation (RFA), laser ablation, high intensity focused ultrasound (HIFU), and microwave ablation. The RFA in kidney tumor treatment was reported with ureteral injury, inflammatory tract mass, and transient lumbar plexus pain [17]. However, IRE is a non-thermal ablation modality that reduces damage to major blood and lymphatic vessels or bile ducts around the tumor tissue in human clinical studies during the ablation process with ECG-synchronized delivery [21,22,23]. In general, immune cells migrate into the tumor through blood or lymphatic vessels. Thus, when combining IRE with immunotherapy, it has the advantage of not interfering the migration of immune cells into the tumor site. In this regard, in recenSt years, the combination of IRE with immunotherapy has been actively researched for the purpose of effective tumor treatment [18,24,25].

The stimulator of interferon genes (STING) is a recently discovered regulator of tumor immunity, which is critical for the innate immune sensing of tumor cells and cross-priming of CD8^+^ T cells [26,27,28,29]. It is a pattern recognition receptor present in the endoplasmic reticulum, which detects the presence of cytosolic DNA as a pathogen-associated molecular pattern [30,31]. When STING signaling is triggered within the tumor microenvironment by tumor cell-derived DNA, it activates dendritic cells, macrophages, and tumor endothelial cells to produce type I interferon through interferon response factor-3 and potentiate the spontaneous priming of tumor-specific T cells [32,33]. Because STING-deficient mice failed to recognize and reject immunogenic tumors, STING signaling seemed to be indispensable for the immune-mediated sensing and control of tumors in vivo [26]. Therefore, the targeting of STING signaling is now considered an essential therapeutic strategy that can convert poorly immunogenic tumors to highly immunogenic tumors, which can respond favorably to cancer immunotherapy [3,31,34]. In this regard, we hypothesized that STING agonists could be an ideal partner for IRE because it can facilitate the innate immune sensing of the tumor antigens and tumor DNA that are massively released from dying tumor cells after IRE.

**Figure 1:**
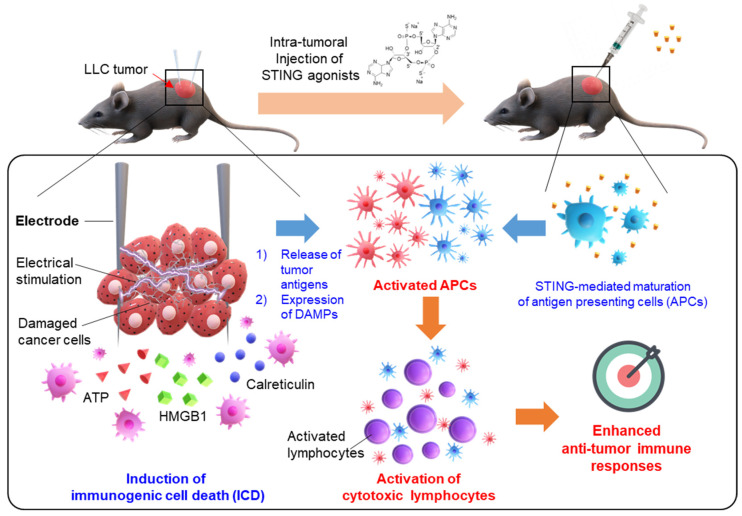
Schematic illustration of a combination of irreversible electroporation (IRE) with STING agonists for effective cancer immunotherapy. IRE induces immunogenic cell death (ICD) of cancer cells, leading to the release of tumor antigens and the expression of damage-associated molecular patterns (DAMPs). Additionally, intratumoral injection of STING agonists induces maturation of antigen-presenting cells (APCs), triggering the activity of cytotoxic lymphocytes. As a result, the combination of IRE and STING agonists can effectively induce anti-tumor immune responses.

In this study, the anti-cancer immune response was effectively elicited by the combination treatment of IRE and STING agonists. We proved that IRE generates tumor antigens and damage-associated molecular patterns (DAMPs) via ICD and intratumoral injection of the STING agonist induces maturation of APCs, ultimately activating cytotoxic T lymphocytes to eradicate cancer cells effectively (Figure 1). Moreover, optimal IRE conditions for immunotherapy were determined through in vitro experiments. In addition, in the in vivo tumor model, superior tumor growth inhibition was confirmed by the combination of an IRE and STING agonist.

## 2. Materials and Methods

### 2.1. Finite Element Method (FEM) Simulation

The IRE area was evaluated using finite element analysis (COMSOL Multiphysics 5.4), closely following previous studies [35,36,37]. The ablation area was determined as the total applied electrical energy (w) exceeding a critical value (w_c_) of 5.9 × 10^5^ J/m^3^, as reported previously [36]. The applied electrical energy density w is given by

(1) $$w=E^{2}\cdot n∆t\cdot \sigma$$

where *E* is the electric field, *n* is the number of pulses, ∆*t* is the time duration of the pulse, and *σ* is the electrical conductivity of the tissue. The electric field distribution over the tissue was solved using the Laplace Equation with the time-dependent solver.

The Laplace Equation is as follows:

(2) ∇(σ∇φ)=0

where *φ* is the electrical potential. Circular cancer tissue with a 30 mm diameter was assumed to be electrically homogeneous with a conductivity of 0.125 S/m [37]. A pair of cylindrical electrodes with 0.1 mm diameter was positioned at the center of the tissue, with a 3 mm center-to-center distance to apply IRE pulses; rectangular pulses with a 1000 V voltage were applied between two electrodes with a 100 μs pulse duration. The ablation area was calculated for each applied pulse number of 8, 40, and 80.

### 2.2. In Vitro Cell Culture

The Lewis lung carcinoma (LLC, CRL-1642) cell line was purchased from ATCC. When the LLC cells reached a density of approximately 70% in a 100 mm culture dish, they were passaged. The cell culture medium was maintained in DMEM containing 10% FBS and 1% penicillin-streptomycin.

### 2.3. In Vitro 3D Tumor Model

For the 3D culture model, a kit based on alginate and gelatin (3D Cell Culture System-24 well kit, MediFab, Seoul, Korea) was used. 3D cell culture was performed using the method provided by the manufacturer. After the casting gel was completely liquefied at 70 °C, the solution (2 mL) was dispensed into 24 wells, and a casting mold was installed to harden at 4 °C for 30 min. The cell dispersion (1 × 10^6^ cells/mL) and the Bio-gel solution at a 2:8 volume ratio were dispensed at 300 μL each into the grooves of the completely hardened casting gel. The bio-gel containing the cells was allowed to stand at 37 °C for 20 min and 4 °C for 10 min to completely gel. After gelation, 3D cells were transferred to a 24 well plate and cultured in 2 mL of culture medium for 24 h for use in further experiments.

### 2.4. In Vitro IRE Treatment

Electroporation was performed using an electroporation system (BTX Harvard Apparatus, Holliston, MA, USA) with a specified voltage, pulse duration, and number of repetitive pulses (voltage: 1000 V, pulse duration: 100 μs, number of repeated pulses: 8, 40, and 80). The cells were suspended in PBS at 1 × 10^6^ cells/mL and then electroporated at room temperature by direct contact with a plate electrode with a space of 4 mm. After electroporation, the cell suspension was kept on ice and biologically analyzed within 30 min. Alternatively, the electroporated cells were transferred to the culture dish and cultured for 24 h before analysis.

### 2.5. In Vitro Cell Viability Analysis

In vitro cell viability was measured using the D-Plus™ CCK cell viability assay kit (CCK-3000, DOJINDO, Kumamoto, Japan) and LIVE/DEAD^®^ Viability/Cytotoxicity Kit (L3224, Invitrogen, Carlsbad, CA, USA).

To measure cell viability using CCK-8, cells (1 × 10^5^ cells/mL) treated with IRE were seeded in 96 plates. After 24 h, CCK-8 solution (10 μL) was added to each well, followed by incubation at 37 °C for 2 h. Absorbance was measured at 450 nm using a microplate reader. Cell viability was expressed as a percentage compared to the control (untreated). After electroporation under various conditions, the average absorbance value from 8 wells was measured.

To perform the LIVE/DEAD assay, after electroporation on the 3D tumor model, Calcein AM and EthD-1 were mixed and incubated for 1 h at room temperature. Survival cells were stained with Calcein AM and dead cells with EthD-1. Finally, the sample was imaged using fluorescence microscopy and quantitative analysis was performed using ImageJ software (Image J 1.52a USAJAVA 1.8.0_112 (64-bit), the National Institutes of Health, Bethesda, MD, USA).

### 2.6. In Vitro Analysis of the Expression of DMAPs

To prepare the cancer cells for observation with the confocal microscope, the cells were electroporated in DMEM under various conditions in a 4 mm spaced cuvette type electrode [38,39]. A coverslip (25 mm) was placed on a 6 well plate, and 1 × 10^6^ cells/well were incubated for 24 h. The coverslips were washed with PBS and then fixed with 4% paraformaldehyde (PFA) for 20 min at room temperature. To improve the intracellular permittivity of Phalloidin, 0.1% Triton X-100 was treated with cells for 5 min, and Phalloidin (A12381, Invitrogen, CA, USA) and Calreticulin (CRT, NBP1-47518AF488, Novus, Centennial, CO, USA) were stained for 20 min and 1 h, respectively. The DAPI solution was dropped on the cells, and the coverslip was mounted on the slide. Samples were imaged using an LSM 880 microscope (Zeiss, Carl Zeiss AG, Oberkochen, Germany) and the images were quantitatively analyzed using Image J software.

The supernatant obtained after treatment with IRE on the cells was quantified using ELISA for HMGB1 (NBP2-62767, Novus, CO, USA) and ATP (FLAA-1KT, Sigma, St. Louis, MO, USA) expression levels. The analysis was performed according to the manufacturer’s instructions.

### 2.7. In Vivo Tumor Treatment with a Combination of IRE and STING Agonist

The animal experiments were approved by the Institutional Animal Care and Use Committee (IACUC) (IACUC Approval Number 180103). Seven-week-old C57BL/6 mice were purchased from Orient Bio (Gyeonggi-do, Korea). All experiments were conducted after one week of adaptation. Mice were housed in a breeding environment that maintained a 12 h night and day cycle, at a temperature of 20–24 °C and humidity of 44.5–51.8%.

To generate tumor models, LLC (5 × 10^5^ cells) were subcutaneously injected into C57BL/6 mice. During the treatment, tumor size and body weight were measured every two days. Tumor-bearing mice were randomized to either (1) Non-treated group (Control, *n* = 5), (2) IRE-treated group (IRE, *n* = 5), (3) STING agonist-treated group (STING, *n* = 5), or (4) IRE plus STING agonist-treated group (IRE/STING combination, *n* = 5). Intraperitoneal anesthesia was performed using the IACUC approved ketamine and xylazine cocktails.

The anesthetized mice were electroporated using a two-needle array electrode with a 3 mm spacing made of medical-grade stainless steel. When the tumor grew to a size of 3 mm (diameter), the needle electrode was inserted entirely into the center of the tumor and then electroporated. Electroporation was applied under the conditions determined in the in vitro cell experiments (voltage: 1000 V, pulse duration: 100 μs, pulses: 40). Following IRE according to a pre-planned schedule, the STING agonist (10 μg) was injected twice in the intra-tumoral route (Figure 4b).

### 2.8. Tumor-Infiltrating Immune Cell Analysis Using Flow Cytometry

For flow cytometry analysis, tumors were harvested and dissociated into a single-cell suspension by 1-h incubation at 37 °C in digestion buffer containing 2 mg/mL collagenase D (COLLD-RO, Merck, Kenilworth, Darmstadt, Germany) and 40 μg/mL DNase I (10104159001, Merck). The cell clumps were removed through a 70-μm cell strainer (352350, Corning, NY, USA) and a 40-μm nylon mesh. The red blood cells were removed by incubation for 3 min at RT in ACK lysis buffer (A1049201, Fisher Scientific, Waltham, MA, USA). To exclude dead cells, the dissociated cells were stained with Fixable Viability Dye eFluorTM 450 (65-0863-18, Invitrogen) on ice for 30 min before antibody staining. After washing with FACS buffer (1% fetal bovine serum in PBS), the cells were incubated on ice for 30 min in FACS buffer with the following antibodies: FITC-conjugated anti-CD11b (rat, clone M1/70, Invitrogen), FITC-conjugated anti-CD4 (rat, clone RM4-5, Invitrogen), Alexa Fluor 488-conjugated anti-mouse iNOS (rat, clone CXNFT, Invitrogen), PE-conjugated anti-mouse CD8a (rat, clone 53-6.7, Invitrogen), PE-conjugated anti-mouse CD86 (rat, clone B7-2, Invitrogen), PE-conjugated anti-mouse F4/80 (rat, clone BM8, Invitrogen), PerCP/Cy5.5-conjugated anti-mouse Ly-6C (rat, clone HK1.4, Invitrogen), PerCP/Cy5.5-conjugated anti-mouse CD45 (rat, clone 30-F11, Invitrogen), APC-conjugated anti-mouse CD3 (rat, clone 17A2, Invitrogen), APC-conjugated anti-mouse CD11c (armenian hamster, clone N418, Invitrogen), APC-conjugated anti-mouse Ly-6G (rat, clone 1A8-Ly6g, Invitrogen), Alexa Fluor 700-conjugated anti-mouse Arginase 1 (rat, clone A1exF5, Invitrogen), APC/eFluor 780-conjugated anti-mouse CD11b (rat, clone M1/70, Invitrogen), APC/Fire750-conjugated anti-mouse CD80 (armenian hamster, clone 16-10A1, BioLegend), or eFluor 506-conjugated anti-mouse CD45 (rat, clone 30-F11, Invitrogen). We analyzed the following cell subsets: M1-like TAMs, gated as viability dye^−^/CD45^+^/CD11b^+^/Ly-6G^−^/Ly-6C^−^/F4/80^+^/iNOS^+^/Arg1^−^ cells; M2-like TAMs, gated as viability dye^−^/CD45^+^/CD11b^+^/Ly-6G^−^/Ly-6C^−^/F4/80^+^/iNOS^−^/Arg1^+^; TILs, gated as viability dye^−^/CD45^+^/CD3^+^/CD8^+^ or CD4^+^ cells; activated DCs, gated as viability dye^−^/CD45^+^/CD11c^+^/CD86^+^ or CD80^+^ cells. The stained cells were analyzed using a CytoFLEX flow cytometer (Beckman Coulter, Brea, CA, USA), and the data were analyzed with FlowJo software (Tree Star, San Carlos, CA, USA).

### 2.9. Statistical Analysis

All studies were performed independently based on three or more repetitions. Statistical significance was determined by one-way *t*-test performed in Prism statistical software (Version 5, GraphPad, La Jolla, CA, USA). When the *p*-value was less than 0.05, it was judged that there was statistical significance.

## 3. Results and Discussion

### 3.1. In Vitro Cellular Experiments to Establish Electrical Conditions for IRE

**Figure 2:**
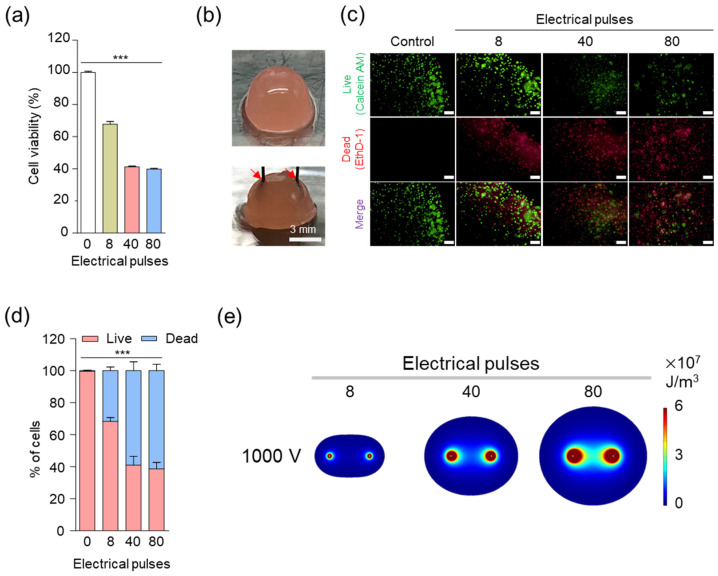
In vitro cellular experiments to establish electrical conditions for irreversible electroporation (IRE). (**a**) In vitro cytotoxicity of LLC under various electrical pulses in IRE (voltage: 1000 V). Asterisks indicate statistical significance compared to the control group (*** *p* < 0.0001, *n* = 3). (**b**) Digital photograph of the 3D tumor model. LLC cells were cultured in a hydrogel containing alginate and gelatin, and a needle electrode with a 3 mm spacing was used for the IRE. The red arrow indicates the electrode. (**c**) In vitro live and dead cell viability assay of LLC under various electrical pulses in the IRE (at 1000 V). Live and dead cells were stained with Calcein AM (green) and propidium iodide (red), respectively (scale bars = 200 μm). (**d**) Quantitative analysis of the live and dead cell assay results. Asterisks indicate statistical significance compared to the control group (*** *p* < 0.0001, *n* = 3). (**e**) Finite element method (FEM) simulation images of IRE area. The color indicates the electric energy density level of the IRE zone with 8, 4, and 80 pulses (voltage: 1000 V and pulse duration: 100 μs).

IRE generally ablates tumor tissues by damaging the cellular membrane using the specific conditions of voltage (1000 V), duration (100 μs), and 8 pulses [14,40]. However, since voltages above 1000 V showed too much toxicity in vitro condition (data not shown), we evaluated the cancer cell death rate by fixing the voltage at 1000 V and changing the pulses. Cancer cells were cultured in two dimensions in vitro to perform IRE under various pulse conditions and confirm the viability of LLC tumor cells by CCK-8 assay (Figure 2a). The cell death rate increased as the number of pulses increased, but no significant change in cell death rate was observed when more than 40 pulses were applied. Since 2D cell culture does not represent in vivo 3D tumor tissue, it is difficult to evaluate the effect of treatment with IRE accurately. To mimic in vivo conditions and evaluate cell death by IRE, an in vitro alginate-gelatin 3D-cell culture model (Figure 2b) was prepared and applied with IRE with a 3 mm two-needle array [41,42,43]. Live and dead assays were performed 24 h after applying 8, 40, and 80 pulses at 1000 V, which are the same conditions as the 2D experiment. As shown in Figure 2c, as the number of pulses increased, live cells (Calcein AM, green color) decreased, and dead cells (EthD-1, red color) increased. Quantification of the fluorescence signal of microscopic images (Figure 2d) revealed that the death rate increased as the number of pulses increased, but there was no significant difference in the cell death rate above 40 pulses. The results of 3D culture were consistent with those in 2D culture. These results indicate that cancer cells can be effectively destroyed with 40 pulses at a voltage of 1000.

We used COMSOL to observe the IRE area when a two-needle type with a spacing of 3 mm and a thickness of 0.1 mm was applied to a spherical cancer tissue with a diameter of 30 mm (Figure 2e). The ablation zone of the simulation indicates the ablation area for 8, 40, and 80 pulses at 1000 V, as in the above cell experiments. The ablation area increased significantly with increasing pulse parameters [36]. This result means that although IRE can affect normal tissues, it can be accurately controlled by predicting the ablation zone through the computer simulation.

### 3.2. In Vitro Immunogenic Cell Death Induced by IRE

Immunogenic cell death (ICD) in the tumor microenvironment (TME) exposes damage-associated molecular patterns (DAMPs) to mature antigen-presenting cells (APCs) such as dendritic cells (DCs), resulting in anti-cancer immunity [44]. ICD triggered by IRE has recently been reported [18,20,44,45]. Generally, it has been reported that IRE induces apoptosis of cancer cells [18,46]. Expression of DAMP markers by ICD stimulates antigen-presenting cells and cytotoxic T lymphocytes, thereby maximizing anti-tumor immunity against cancer cells [47,48]. The major DAMPs markers are CRT, HMGB1, and ATP [9,49].

**Figure 3:**
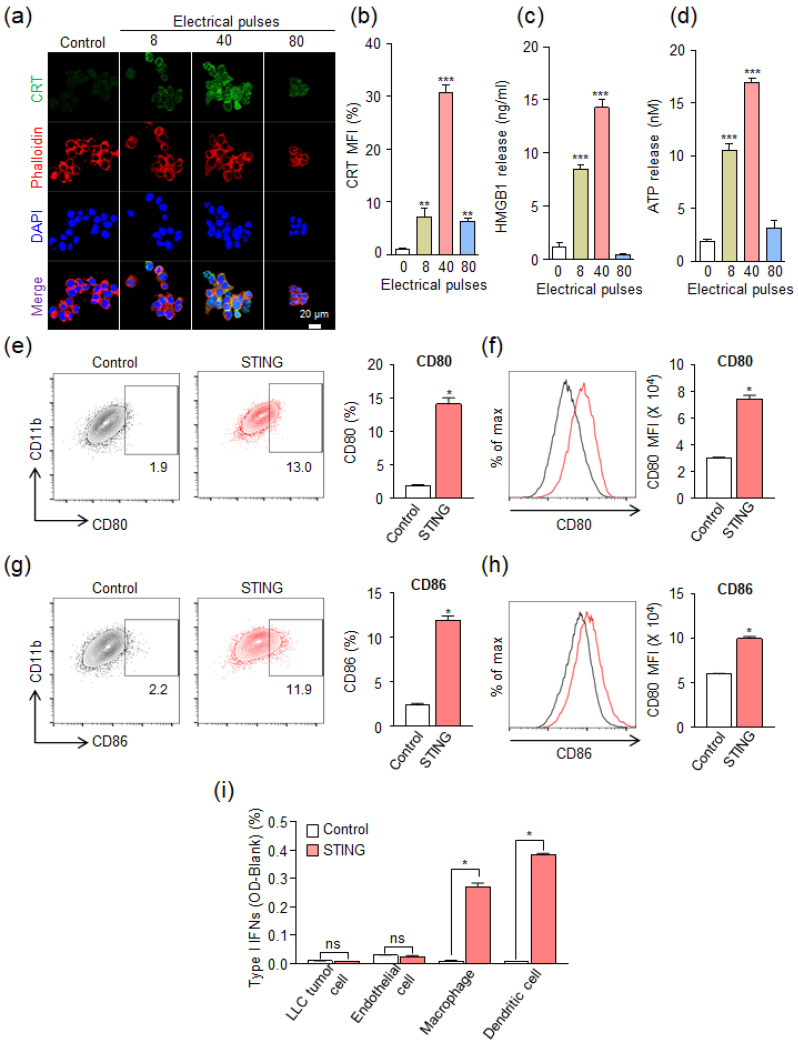
In vitro immunogenic cell death (ICD) was induced by irreversible electroporation (IRE). (**a**) Expression of calreticulin (CRT) on the surface of the LLC after IRE under various electrical pulses at a voltage of 1000 V. Confocal microscopy images were obtained 24 h after IRE. (**b**) Quantitative analysis of CRT expression levels in the confocal microscopy results compared to the control group (** *p* < 0.01, *** *p* < 0.0001). (**c**) Amount of released HMGB1 from LLC at 24 h after IRE under various electrical pulses at a voltage of 1000 V (*** *p* < 0.0001). (**d**) Amount of release ATP from LLC immediately after IRE under various electrical pulses at a voltage of 1000 V (*** *p* < 0.0001). (**e**–**h**) Flow cytometry analysis of the murine myeloid cells (RAW264.7) at 24 h after treatment STING agonist (RR-CDA, 10 μg/mL). Representative flow cytometric plots and quantitative analysis of (**e** and **f**) CD80^+^ and (**g** and **h**) CD86^+^ cells (* *p* < 0.05). (**i**) Type-I IFNs activation after STING agonist treatment (RR-CDA, 10 μg/mL) in various cell types. In Figure 3e–i, control means a non-treated group. Pooled data from two independent experiments with *n* = 5 per group. Values are mean ± SD. Two-tailed Student *t* test.

To evaluate the expression of the DAMP signal induced by IRE, CRT, HMGB1, and ATP were analyzed after IRE treatment of cancer cells at 8, 40, and 80 pulses at 1000 V. In Figure 3a, the highest CRT expression was shown at 40 pulses, but at 80 pulses, the expression pattern was similar to that of 8 pulses. When quantitatively compared with the untreated control group (Figure 3b), it was confirmed that CRT expression was increased by about 30 times at 40 pulses. It increased slightly by 6.9 times at 8 pulses and 6.1 times at 80 pulses. The expression of HMGB1 compared to the control group increased by 7.3 times at 8 pulses and 12.3 times at 40 pulses, but did not increase at 80 pulses (Figure 3c). ATP expression compared to the control group was increased by 5.4 times at 8 pulses, 8.7 times at 40 pulses, and 1.6 times at 80 pulses (Figure 3d). However, the STING agonist did not show a significant difference in the expression of CRT and HMGB1 compared to the control group (Figures S1 and S2), which indicates that the STING agonist itself does not induce ICD. In summary, the expression of markers of DAMPs signal was observed under an IRE condition of 40 pulses, but relatively weak expression was observed at 8 pulses and 80 pulses. These results indicate that there is an optimal electrical pulse condition that is neither too weak nor too strong to express DAMPs by ICD. It is speculated that low pulses (<40) do not induce ICD, and high pulses (>40) induce rapid cell death, limiting the expression of DAMPs. Through these experimental results, it was found that DAMP markers were significantly expressed in the electrical condition of 40 pulses at 1000 V. Based on the experimental results of cell viability and DAMP expression in various electrical pulses, the electrical condition of 40 pulses at 1000 V (pulse duration: 100 μs) was selected for IRE in further experiments.

To investigate whether treatment with a STING agonist can activate APCs, we investigated the expression of maturation markers after STING agonist treatment on murine myeloid cells (RAW264.7) (Figure 3e–h). STING agonists are known to be potent immune adjuvants that can link immunogenic cell death to innate immune sensing of tumor antigens [26]. In this study, we selected RR-CDA as a combination partner for IRE because it can more strongly enhance innate and adaptive cancer immunity compared to canonical STING agonists. Especially, the activation of APCs was more potent with RR-CDA compared to cGAMP [33]. Moreover, it is more clinically feasible because it can activate all allelic variants of human STING protein as well as mouse STING protein, while others cannot [50]. Interestingly, STING agonist treatment dramatically elevated CD80 expression (Figure 3e,f) and CD86 (Figure 3g,h) in RAW264.7 cells, compared to the control. This result indicates that the STING agonist can effectively activate APCs. Additionally, we used a Type-I interferon reporter assay to measure Type-I interferon activity following STING agonist treatment (Figure 3i). RR-CDA remarkably induced Type-I interferon activation in macrophages and dendritic cells, while there are no changes in tumor cells and endothelial cells. Taken all together, the expression of DAMPs through the induction of IRE-mediated ICD and activation of APCs by STING agonists implies that all of these techniques can be involved in the anti-tumor immune responses.

### 3.3. In Vivo Tumor Suppression by Combinational Treatment with IRE and STING Agonist in an LLC Tumor Model

**Figure 4:**
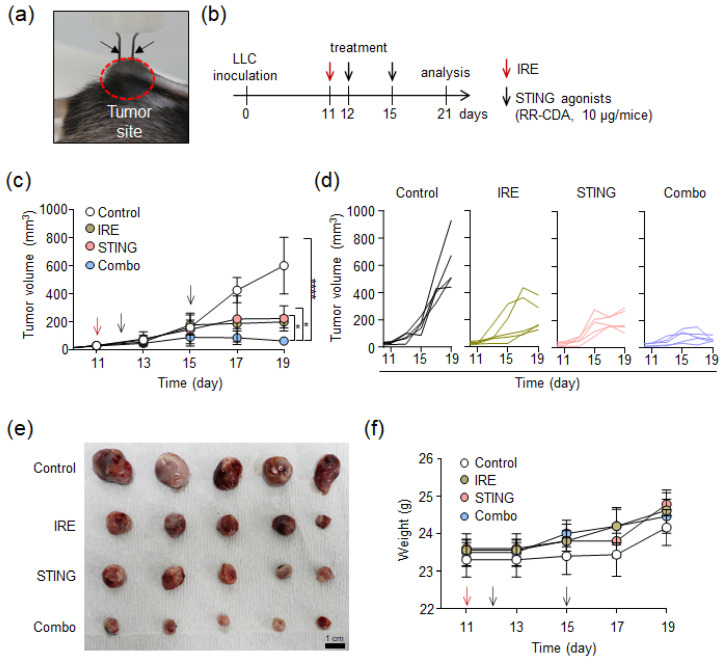
In vivo tumor suppression by combinational treatment of irreversible electroporation (IRE) and STING agonist in the LLC tumor model. (**a**) Digital photograph of two-needle electrodes injected into LLC tumor-bearing C57BL/6 mice. The black arrow indicates the electrode. (**b**) In vivo experimental schedule for the combination treatment of IRE (voltage: 1000 V, pulse duration: 100 μs, pulses: 40) with STING agonist (RR-CDA, 10 μg) after the inoculation of LLC cells into C57BL/6 mice. The IRE and STING agonists were treated at the described time points. (**c**) Mean change in tumor growth of mice after combinational treatment with IRE and STING agonists (*n* = 5, * *p* < 0.05, **** *p* < 0.00005). (**d**) In vivo individual tumor growth curve after the combinational treatment of IRE with STING agonists (*n* = 5 for each group). (**e**) Digital photographs of tumors extracted after treatment for each group. (**f**) Mean change in body weight of mice during treatment for each group (*n* = 5).

To confirm whether the IRE and STING agonist treatments cooperate to establish a robust anti-tumor immunity in TME, we treated LLC tumor-bearing mice with IRE and/or a STING agonist (Figure 4). When the tumor size reached more than 3–4 mm in the longest diameter, 2 needle-shaped electrodes (3 mm apart) were inserted into the tumor to perform IRE (Figure 4a). As depicted in Figure 4b, LLC tumors reached 3–4 mm in diameter on day 11 after tumor injection. After IRE on day 11, the STING agonist (10 μg/mice) was injected twice on days 12 and 15, respectively. The optimal time interval between first and subsequent STING agonist injection was determined from our previous report [33], in which a 2- or 3-day-interval was optimal to fully induce both innate and adaptive immune responses within TME. Finally, on day 21, tumors were harvested for TME analysis.

As shown in Figure 4c, both the IRE and IRE/STING groups showed slow tumor growth compared to the control group, but the largest tumor growth inhibition was observed in the IRE/STING combination group. In individual tumor growth curves (Figure 4d), tumors in the control group grew rapidly in all mice. In the IRE-treated group, tumors showed rapid growth in two mice and then decreased, which is presumed to be due to the triggering of anti-tumor immunity through the electric ablation-mediated ICD. In the IRE/STING combination group, tumors were almost regressed in all mice on day 21. After the in vivo experiment, tumor masses were completely resected and photographed with a digital camera (Figure 4e). In the IRE/STING combination group, tumor size was significantly smaller in contrast to the other groups. In addition, there were no differences in body weight among all groups and no gross abnormalities during treatment (Figure 4f). These combined results suggest that the combination of IRE and STING agonists can effectively inhibit tumor growth without significant adverse events.

### 3.4. Induction of Anti-Cancer Immunity in TME by a Combination of IRE and STING Agonists

**Figure 5:**
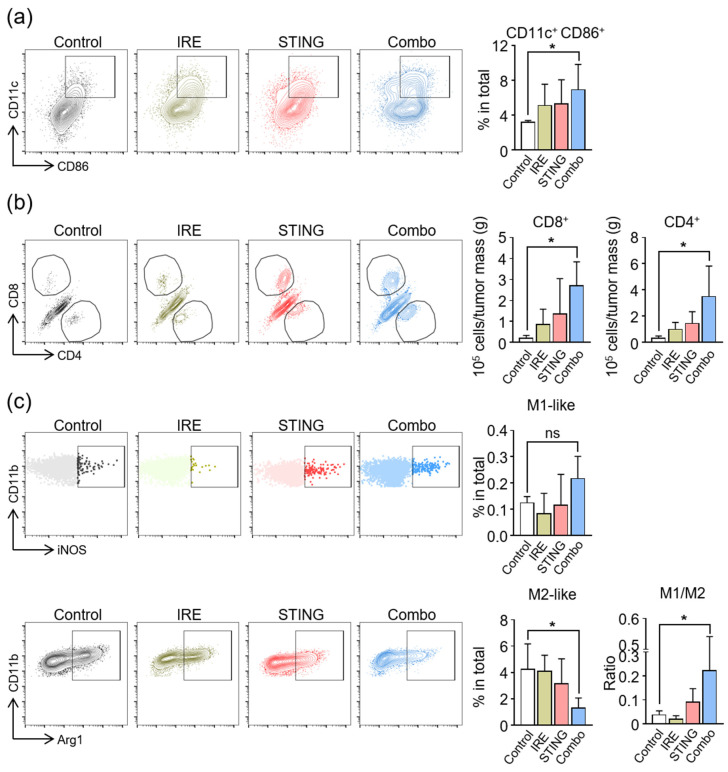
Combination treatment with irreversible electroporation (IRE) and STING agonist remodels the composition of immune cells in the tumor microenvironment (TME). Intratumoral CD45^+^ immune cells were analyzed by flow cytometry (* *p* < 0.05). (**a**) Representative flow cytometric plot and comparisons of CD86^+^CD11c^+^ activated DCs. (**b**) Representative flow cytometric plot and comparisons of the cell numbers of CD8^+^ T cells and CD4^+^ T cells (* *p* < 0.05). (**c**) Representative flow cytometric plot and comparisons of CD11b^+^F4/80^+^iNOS^+^ M1-like macrophages, CD11b^+^F4/80^+^Arg1^+^ M2-like macrophages, and M1/M2 ratio (* *p* < 0.05). Pooled data from two independent experiments with *n* = 5 per group. Values are mean ± SD. Two-tailed Student *t* test.

To investigate the immunologic changes within TME, which were elicited by IRE and STING agonist treatment, we analyzed innate and adaptive immune cells within tumors by flow cytometry. The combination treatment markedly increased the number of activated DCs (CD86^+^CD11c^+^ cells) by 2.2 times, whereas IRE or STING agonist monotherapy did not significantly activate DCs when compared to the controls (Figure 5a). Moreover, combination treatment increased the intratumoral infiltration of CD8^+^ cytotoxic T cells by 11.2 times and that of CD4^+^ T cells by 6.4 times compared to the control group (Figure 5b). Since macrophages are another important player of innate immunity within TME, and STING signaling is involved in the activation and repolarization of macrophages [33,51,52], we also analyzed tumor-associated macrophages (Figure 5c). While IRE alone did not significantly affect the phenotypes of macrophages, STING agonist monotherapy marginally increased the M1/M2 macrophage ratio. This macrophage repolarization was most evident when tumors were treated with the combination of IRE and STING agonists, where M1-like macrophages increased by 1.7 times compared to control and M2-like macrophages decreased by 70% compared to the control, thereby resulting in a 5.9-fold increase in M1/M2 ratio within TME, compared to control tumors.

In the present study, IRE and STING agonist treatments complement each other to enhance anti-cancer immunity at multiple levels. First, IRE activates DCs by inducing immunogenic cell death within the tumor microenvironment. Since STING signaling is also involved in this process [30,31,33,34], the combined STING agonist treatment may have a positive impact on this IRE-induced DC activation. Second, IRE fortified STING-induced macrophage repolarization toward the anti-tumor M1 phenotype. Although STING agonist monotherapy marginally increased the M1/M2 ratio within TME, the combination treatment of the IRE and STING agonist strongly induced a favorable M1/M2 macrophage balance within TME. Lastly, the combination of IRE and STING agonists strongly elicit a robust adaptive immune response by accumulating CD8^+^ or CD4^+^ effector T cells into the TME. This may be the result of enhanced T cell priming by activated DCs stimulated with an IRE and STING agonist. Moreover, STING agonists can facilitate the intratumoral infiltration of activated T cells because they are known to remodel tumor vasculatures suitable for lymphocyte extravasation [33]. Our findings indicate that the combination treatment of IRE and STING agonists effectively induced strong anti-tumor immunity by remodeling innate and adaptive immune cells within the TME. The adaptive immunity triggered by the combinational treatment of IRE and STING will be conducted through intracellular cytokine staining, ELISPOT, or assessment of abscopal or memory effects, referring to recent studies [53,54], in future studies.

## 4. Conclusions

In this study, the combination of irreversible electroporation and STING agonists was evaluated to induce effective anti-cancer immune effects. Optimal IRE conditions (i.e., voltage: 1000 V, pulse duration: 100 μs, pulses: 40) were determined through in vitro 2D and 3D cell culture. Under these conditions, IRE effectively induced ICD by expressing CRT, HMGB1, and ATP. Additionally, the combination of IRE and STING agonists effectively matured DCs. In the in vivo tumor growth inhibition test, the IRE/STING combination group significantly reduced tumor size compared to the IRE or STING agonist treatment group. In the IRE/STING combination group, the number of activated DCs in the tumor increased, and the number of CD8^+^ and CD4^+^ cells also increased. In addition, the combination treatment reduced the number of M2-like macrophages and increased the number of M1-like macrophages in the tumor tissues. These results suggest that the combination of ICD with an IRE and STING agonist, an immune adjuvant, can effectively trigger anti-tumor immunity within TME. In particular, this strategy could be used to develop a potent tumor vaccine by effectively inducing the expression of tumor-specific antigens. Detailed animal experiments and immunoassays are currently in progress. We believe that the combination of IRE and STING agonists may be a promising therapeutic strategy for poorly immunogenic refractory cancers.

## Supplementary Materials

The following are available online at https://www.mdpi.com/2072-6694/12/11/3123/s1, Figure S1: Expression of calreticulin (CRT) on the surface of LLC after STING agonist (RR-CDA) treatment. Figure S2: Quantitative analysis of HMGB1 released from LLC after STING agonist (RR-CDA) treatment.
