# Supplementary material for: Combination of Irreversible Electroporation and STING Agonist for Effective Cancer Immunotherapy
Source: Cancers (Basel). 2020 Oct 26;12(11):3123. doi: 10.3390/cancers12113123 (PMC7693597; doi:10.3390/cancers12113123)
Supplement: Supplementary file 1 [file cancers-12-03123-s001.pdf]

cancers

MDPI

Supplementary Materials

Combination of Irreversible Electroporation and STING Agonist for Effective Cancer Immunotherapy

Eun-Jin Go, Hannah Yang, Hong Jae Chon, DaSom Yang, WonHyoung Ryu, Dong-Hyun Kim, Dong Keun Han, Chan Kim and Wooram Park

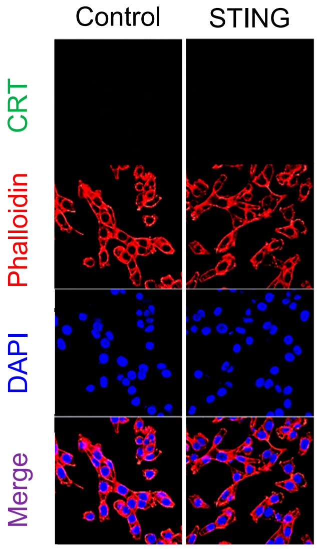

Figure S1. Expression of calreticulin (CRT) on the surface of LLC after STING agonist (RR-CDA) treatment. STING agonist was treated at a concentration of 10 μg/mL. Confocal microscopy images were obtained 24 h after the STING agonist treatment.

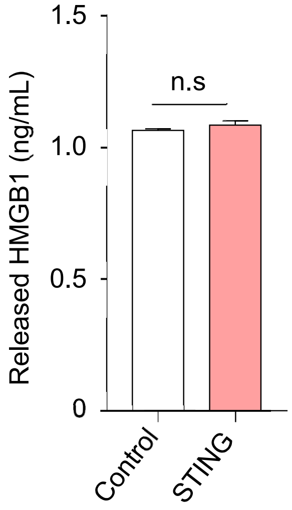

Figure S2. Quantitative analysis of HMGB1 released from LLC after STING agonist (RR-CDA) treatment. To measure the amount of released HMGB1, LLC cells were treated with STING agonist (10 μg/mL) in 24 well plates for 24 h. The released HMGB1 was quantified using ELISA at 450 nm.
